# A Prospective Randomised Pilot Study on the Timing of Contrast Media Administration in Adhesive and Virgin Abdomen Small Bowel Obstruction

**DOI:** 10.3390/medicina62050998

**Published:** 2026-05-20

**Authors:** Liis Jaanimäe, Urmas Lepner, Ülle Kirsimägi, Virve Saarevet, Ceith Nikkolo

**Affiliations:** 1 Surgery Clinic, Tartu University Hospital, Puusepa 8, 51014 Tartu, Estonia; 2 Faculty of Medicine, University of Tartu, Ravila 19, 50411 Tartu, Estonia; 3 Division of Acute Care Surgery, North Estonia Medical Centre, Sütiste tee 19, 13419 Tallinn, Estonia

**Keywords:** small bowel obstruction, adhesions, contrast media, Estonia, general surgery

## Abstract

*Background and Objectives:* Small bowel obstruction (SBO) is a common surgical emergency, accounting for 15–20% of acute general surgical admissions. Despite the Bologna Guideline’s introduction to the surgical community almost a decade ago, adherence to it remains variable. The therapeutic role of contrast media and the optimal timing of its administration remain a matter of debate. This study aimed to compare SBO resolution rates according to the timing of water-soluble contrast media (WSCM) administration. *Materials and Methods:* A prospective, randomised pilot trial was conducted at two regional hospitals in Estonia. Patients hospitalised with adhesive or virgin abdomen SBO were randomised to receive WSCM at either 4 h or 24 h after admission. *Results:* A total of 128 patients were enrolled, with 63 assigned to the 4 h group and 65 to the 24 h group. SBO resolved with conservative management in 74.6% of patients in the 4 h group and 73.8% in the 24 h group. Rates of surgical intervention and bowel resection due to necrosis were comparable between groups. Univariable and multivariable analyses showed no significant association between early administration and improved resolution. A prior history of SBO was associated with a higher likelihood of successful non-operative management. *Conclusions:* conservative management of SBO is safe and effective, and early WSCM administration did not provide a clear additional benefit in this cohort with respect to resolution or surgical outcomes. A prior history of SBO was associated with a higher likelihood of successful conservative management in this cohort. Larger multicentre studies are warranted to further define the optimal timing of contrast administration and to compare isotonic and hyperosmolar agents with respect to clinical outcomes.

## 1. Introduction

Small bowel obstruction (SBO) is a frequently encountered surgical emergency responsible for 15–20% of acute general surgical admissions [1].

Kaplan et al. reported that patients with adhesive small bowel obstruction (ASBO) managed according to the Bologna Guidelines had improved outcomes [2]. The World Society of Emergency Surgery (WSES) recently re-evaluated the previously suggested surgical management of SBO in patients without prior abdominal operations and recommended treating VA-SBO according to the Bologna Guidelines [3,4].

Despite these guidelines, there is still no consensus regarding the optimal timing of contrast media (CM) administration or the number and interval of radiographs during contrast follow-through [2]. Studies evaluating the therapeutic effect of CM have reported conflicting results. A multi-institutional study by Zielinski et al. suggested that the use of Gastrografin was independently associated with successful nonoperative management [5]. However, the therapeutic effect of hyperosmolar contrast agents remains uncertain, as most reviews have not demonstrated a reduction in the need for surgery, as stated by Elsolh et al. [6] In our setting, isotonic water-soluble contrast is used; however, the timing of administration varies and is determined by the treating surgeon. In a previous retrospective study including 1118 patients, we observed that CM administration, particularly within 12 h of admission, may be associated with improved outcomes [7]. A meta-analysis by Koh et al. concluded that CM is useful in guiding decision-making but does not provide an independent therapeutic benefit [8]. More recently, Quach et al. emphasised that, despite multiple randomised trials and meta-analyses, further standardised studies are required to clarify the role of contrast media in the management of SBO [9].

This study aimed to compare the resolution of adhesive and virgin abdomen small bowel obstruction according to the timing of WSCM administration. A secondary aim was to identify factors associated with successful conservative treatment.

## 2. Materials and Methods

This study was designed as a prospective pilot trial to evaluate feasibility and estimate effect sizes for future adequately powered studies. Therefore, no formal sample size calculation was performed. Patients hospitalised at Tartu University Hospital and the North Estonia Medical Centre between September 2023 and March 2025 with adhesive or virgin abdomen small bowel obstruction (VA-SBO) were invited to participate in the study. The inclusion of patients with VA-SBO reflects real-world clinical practice and enhances the study’s external validity. This approach is supported by both the WSES position paper and our previous retrospective study, which demonstrated the safety of conservative management in this subgroup when conducted under appropriate clinical supervision [4,10]. The diagnosis of SBO was based on patient history, clinical examination, and radiological evaluation. Radiological evaluation was performed in all patients, most commonly using computed tomography (CT), which is the imaging modality of choice in both participating centres [11]. All included patients provided written informed consent. Eligible patients were aged 18 years or older.

Patients with paralytic ileus or a history of abdominal surgery within 4 weeks prior to admission were excluded. Patients presenting with signs suggestive of bowel ischaemia were excluded, as the study focused on conservative management. Patients requiring immediate surgery were treated accordingly and were not enrolled in the study.

**Figure 1:**
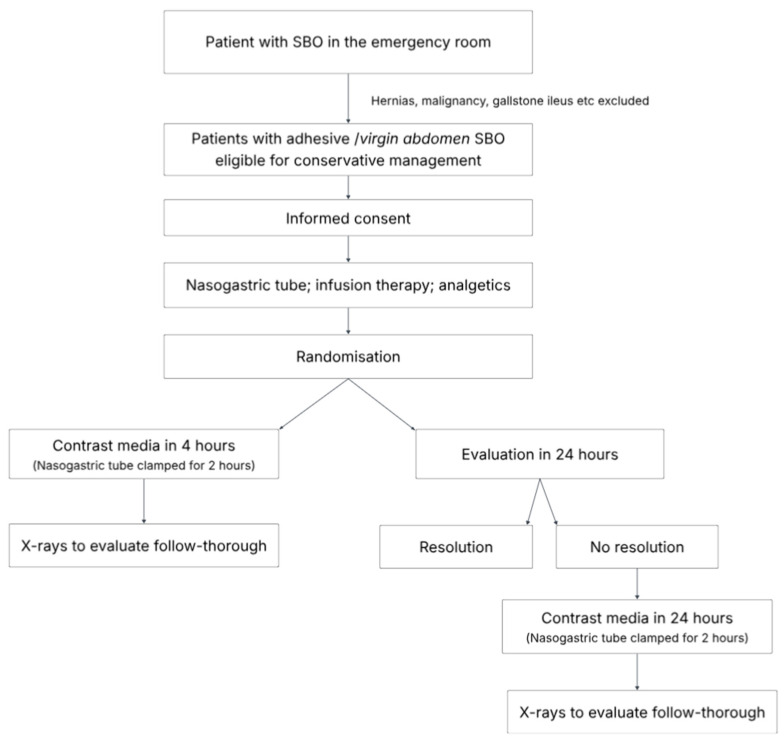
Flow chart describing study protocol.

In both participating centres, patients with suspected SBO are assessed promptly, and conservative management is typically initiated shortly after admission. However, exact emergency department waiting times and time to initiation of treatment were not systematically recorded. Patients were randomised to one of two parallel study groups (Figure 1). Randomisation was performed using sealed, opaque envelopes prepared by one investigator (LJ) prior to study initiation. The envelopes were kept in an arranged location in both hospitals. After obtaining consent, the surgeon or surgical resident on call opened a randomly selected sealed envelope that indicated the patient’s assigned group. Patients were admitted to the surgical ward, and conservative treatment was initiated, including intravenous fluid therapy, analgesia, and nasogastric tube decompression.

Randomisation determined the timing of CM administration: either 4 h or 24 h after admission. In the 4 h group, 100 mL of undiluted WSCM (Visipaque™) was administered via the nasogastric tube after 4 h of decompression. The nasogastric tube was then clamped for 2 h and subsequently reopened. The timing of radiographs was determined by the treating surgeon.

In the 24 h group, patients were evaluated 24 h after admission. If SBO had clinically resolved (defined as passage of gas or stool), no CM follow-through was performed. If signs of SBO persisted, 100 mL of undiluted WSCM (Visipaque™) was administered in the same manner as in the 4 h group.

Resolution of SBO was defined as either clinical resolution (passage of stool or flatus) or radiological resolution (passage of contrast into the colon). These outcomes were not systematically differentiated in all cases, reflecting real-world clinical practice.

Failure of conservative management was determined by the treating surgeon based on a combination of clinical and radiological findings, including persistent obstruction without progression, worsening abdominal pain, signs of peritonitis, rising inflammatory markers, or imaging findings suggestive of bowel ischaemia. In cases of clinical deterioration during the study period, surgical intervention was performed without delay. The exact time from admission to surgical intervention was not systematically recorded for all patients, which limits detailed analysis of timing-related outcomes.

All data were recorded prospectively by L.J. using predefined variables and standardised data fields to ensure consistency. Data were stored on secure hospital servers. The interval between symptom onset and hospital admission was recorded. Patients’ medical history, including previous abdominal operations and prior SBO episodes, was documented. Laboratory results at admission and the number of radiographs performed during follow-through were recorded. In cases of failed conservative management, intraoperative findings (viable bowel, reversible ischaemia, bowel necrosis) and surgical procedures performed (adhesiolysis, bowel resection) were documented. Statistical analyses were performed by a professional statistician using Statistica version 13.3 (TIBCO Software Inc., Palo Alto, CA, USA).

Continuous variables were non-normally distributed and are therefore presented as medians with interquartile ranges (25–75th percentiles). Categorical variables are presented as counts and percentages. Between-group comparisons for categorical variables were performed using Pearson’s chi-square test or Fisher’s exact test, as appropriate. Continuous variables were compared using the nonparametric Mann–Whitney U test.

Univariable and multivariable logistic regression analyses were conducted to assess the association between clinical variables and successful non-operative management. The variables included contrast medium administration at 24 h, symptom duration, history of prior SBO, age, and sex. In the regression models, age and symptom duration were analysed as binary variables (age > 75 years; symptom duration < 24 h). Results are reported as odds ratios (ORs) with 95% confidence intervals (CIs). A *p*-value < 0.05 was considered statistically significant. Given the pilot nature of the study and limited sample size, the multivariable model should be interpreted cautiously.

## 3. Results

A total of 128 patients were enrolled, with 63 randomised to the 4 h group and 65 to the 24 h group. Baseline characteristics were comparable between groups (Table 1). All randomised patients were included in the analysis according to the intention-to-treat principle. Radiological evaluation was performed in all patients, most commonly using computed tomography (CT), which was the primary diagnostic modality in both centres.

SBO resolved with conservative management in 74.6% of patients in the 4 h group and 73.8% in the 24 h group. There was no significant difference between groups in resolution rates, the need for surgery, or the rate of bowel resection.

There were 54 patients with a history of a previous SBO episode, and 21 of them had been previously operated on due to bowel obstruction. The number of prior surgeries ranged from zero to six. Most previous surgeries had been open operations, and only eight patients had a history of only laparoscopic surgery (appendectomy, hysterectomy, cholecystectomy, or adrenalectomy). 13 patients included in the study had not been previously operated on and were diagnosed with VA-SBO.

### 3.1. 4 h Group

**Figure 2:**
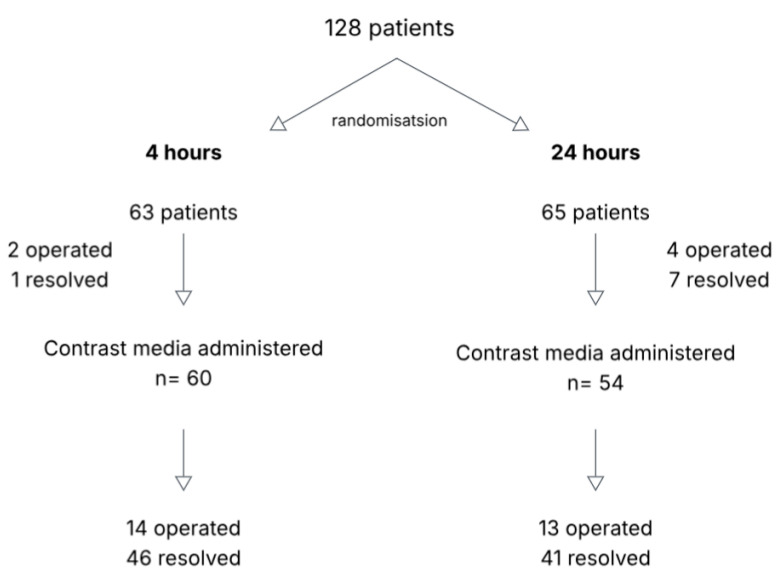
Study groups and outcomes of small bowel obstruction treatment.

In the 4 h group, two patients underwent urgent surgery before contrast administration due to clinical signs suggestive of bowel ischaemia (Figure 2). Surgical exploration demonstrated viable bowel in one patient and reversible ischaemic changes in the other (Table 2). In one patient, the obstruction resolved clinically shortly after admission, and CM follow-through was not performed. In the remaining patients, CM was administered either via a nasogastric tube or orally; nasogastric intubation was not required in seven cases. The number of radiographs obtained ranged from one to four, with a median of two.

Fourteen patients required surgery following failure of conservative management. The duration of conservative treatment varied, with the latest surgical intervention performed on day 4 after randomisation. Intraoperative findings demonstrated viable bowel in seven patients, all of whom underwent adhesiolysis only. Reversible ischaemic changes were observed in five patients, none of whom required bowel resection. Two patients had bowel necrosis requiring resection.

Among operated patients, laparoscopy was performed in six cases, whereas laparotomy was the primary approach in seven cases. In three additional cases, laparoscopy was attempted but had to be converted to laparotomy (Table 2).

### 3.2. 24 h Group

In the 24 h group, four patients underwent surgery before CM administration, while obstruction resolved within 24 h in seven patients (Figure 2). Among patients undergoing early surgery, operative findings demonstrated viable bowel in one case, reversible ischaemic changes in two, and bowel necrosis in one (Table 2). Adhesiolysis alone was performed in three patients, while bowel resection was required in one patient.

CM was administered to the remaining 54 patients. The number of radiographs obtained ranged from one to four, with a median of two, as in the 4 h group. Among these patients, SBO resolved with conservative management in 41 cases, while 13 required surgery. The maximum time to surgical intervention was approximately 70 h from admission.

Among patients undergoing surgery after failed conservative management, viable bowel was observed in seven cases, reversible ischaemic changes in five, and bowel necrosis in one. Adhesiolysis alone was performed in 10 patients, whereas three patients required bowel resection with anastomosis—one due to bowel necrosis and two due to extensive adhesiolysis complicated by serosal injury.

Among operated patients, laparoscopy was successfully performed in six cases, whereas laparotomy was performed in seven cases. In four additional cases, laparoscopy was attempted but required conversion to laparotomy.

Univariable and multivariable logistic regression analyses were performed to assess factors associated with successful conservative management (Table 3).

The analysis demonstrated that patients with a history of SBO were more likely to be successfully treated with conservative management (OR = 3.06; 95% CI [1.23, 7.64], *p* = 0.016).

Timing of CM administration (4 h vs. 24 h), symptom duration < 24 h, age > 75 years, and male sex were not significantly associated with the outcome. Although symptom duration < 24 h and age > 75 years showed a trend toward increased odds of successful conservative management, these associations did not reach statistical significance.

## 4. Discussion

In this prospective randomised pilot study, the timing of WSCM administration (4 h vs. 24 h) did not significantly influence the resolution of small bowel obstruction or the need for surgical intervention. Approximately three-quarters of patients in both groups were successfully managed conservatively, supporting the safety and effectiveness of structured non-operative management in appropriately selected patients.

These findings are consistent with previously reported non-operative success rates of 70–80% in adhesive SBO, supporting the safety and effectiveness of initial conservative management in appropriately selected patients [12,13]. The timing of CM administration did not influence outcomes, including the need for surgery or bowel resection, suggesting that early administration may not be essential for successful conservative management. An important limitation is the use of a composite endpoint for SBO resolution that combines clinical and radiological criteria. These parameters are not equivalent, as radiological passage of contrast into the colon may precede clinical resolution (e.g., passage of stool or flatus). The lack of systematic differentiation between these outcomes may introduce heterogeneity and limit the interpretation of the true clinical recovery.

Delayed surgical intervention has been associated with increased morbidity, mortality, and length of hospital stay [14]. Several predictive models have been developed to identify patients requiring surgical intervention. Maraux et al. proposed a clinical-radiological score that included the age-adjusted Charlson comorbidity index, intestinal obstruction localisation, and the maximum small-bowel diameter/vertical abdominal diameter ratio [15]. Wessmer et al. identified eight variables strongly associated with small bowel resection in their improved scoring system, published in 2023 [16]. In our practice, formal scoring systems are not routinely used, and decisions regarding operative versus non-operative management remain based on clinical judgment. In this cohort, most operated patients had viable bowel or reversible ischaemia, and bowel resection was required in a minority of cases. These findings suggest that timely surgical intervention was achieved when clinically indicated.

Since 1991, when Bastug et al. reported the first laparoscopic adhesiolysis for SBO, the use of laparoscopy for SBO has increased as surgeons gain experience with this technique [17]. In this study, minimally invasive surgery was feasible in a substantial proportion of cases, with laparoscopy successfully performed in nearly half of the operated patients. However, laparoscopy in the setting of distended bowel loops remains technically challenging. Szeliga et al. suggested that laparoscopy could be used mainly in everyday practice for selected cases, such as adhesive SBO caused by single adhesions or foreign bodies in the gastrointestinal tract [18]. Previous studies have shown that laparoscopy may reduce hospital stay, postoperative complications, and mortality compared with open surgery. Additionally, the risks of respiratory, cardiac, and neurological complications, as well as deep vein thrombosis, were significantly reduced after laparoscopic adhesiolysis [19,20]. Farinella’s group reported that the conversion rates from laparoscopy to open surgery vary from 0% to 52%, depending on patient selection and surgical skill [21]. Conversion to laparotomy should not be considered a failure but rather a safe and appropriate intraoperative decision, as Quach and Zielinski state [9]. In our cohort, conversion to laparotomy was required in several patients, reflecting the technical challenges associated with adhesive SBO described above.

The comparable rates of operative intervention and bowel resection between groups suggest that the timing of contrast administration did not adversely affect patient safety.

The similar rates of non-operative resolution observed in both groups suggest that earlier administration of CM may not significantly alter the overall likelihood of successful conservative management, as we hypothesised. The conclusion of our retrospective study suggested that CM may have a therapeutic role in the treatment of SBO, especially when administered within 12 h of admission [7]. Mansoori et al. reported that 85% of the academic radiologic departments in their study used Gastrografin as contrast media [22]. An important aspect of this study is the use of an isotonic water-soluble contrast agent, whereas most previous studies have focused on hyperosmolar agents such as Gastrografin. The proposed therapeutic effect of hyperosmolar contrast agents has been attributed to osmotic fluid shifts, reduction in bowel wall oedema, and stimulation of intestinal motility [23,24,25]. In the present study, resolution rates were comparable to those reported in studies using hyperosmolar agents. These findings suggest that the clinical utility of contrast media may be primarily diagnostic and predictive rather than therapeutic. The passage of contrast into the colon is a well-established predictor of non-operative resolution, and this predictive capability does not appear to depend on the osmolarity of the contrast agent. Furthermore, the use of isotonic contrast media may offer safety advantages, including a reduced risk of fluid shifts, dehydration, and electrolyte disturbances, particularly in elderly or frail patients. The comparable outcomes observed raise the question of whether the osmolar properties of contrast agents are essential for achieving favourable clinical results. It is possible that early administration of any water-soluble contrast agent, combined with standardised clinical management and careful monitoring, plays a more important role than the solution’s specific osmolar characteristics. As most previous trials have evaluated hyperosmolar agents, while this study used an isotonic contrast medium, the findings reflect not only timing but also a different contrast strategy, which may limit direct comparability with existing literature. These findings support the need for further comparative studies to evaluate whether isotonic contrast agents may provide similar clinical benefits while offering a more favourable safety profile.

Survey data indicate considerable variation in the timing of WSCM administration in clinical practice. 28.8% of the surgeons responding to the survey administered CM within 1–12 h of admission, 41.1% within 12–24 h, and 30.1% beyond 24 h of admission [26]. Earlier administration may still offer benefits, such as earlier prediction of resolution and potentially shorter hospital stay [27]. In our cohort, the median length of stay did not differ between study groups.

In this study, a history of prior SBO was associated with a higher likelihood of successful conservative management. The same conclusion was reported by Maienza et al., who found that patients with a history of SBO were more likely to be managed nonoperatively [28]. This may reflect the natural history of recurrent adhesive SBO, which is often caused by single-band or limited adhesions that can produce intermittent or partial obstruction and may resolve spontaneously under conservative management. Previous episodes may also facilitate earlier recognition and presentation, allowing timely initiation of decompression and supportive therapy. However, previous studies have shown that surgical intervention for a first episode may reduce the risk of recurrence (13.0% vs. 21.3%). The risk of additional recurrences increased with each episode until surgical intervention, at which point it decreased by approximately 50% [13].

Key strengths of this study include its prospective randomised design, which reduces selection bias and allows direct comparison of two clinically relevant strategies. The standardised management protocol and detailed documentation of operative findings provide robust and reliable data on the safety of conservative treatment and the timing of surgical intervention. Furthermore, the inclusion of surgical approach and intraoperative findings offers clinically meaningful insight into the feasibility and applicability of minimally invasive surgery in this patient population. An additional strength is that patients were treated at Estonia’s two largest tertiary referral centres, which together manage approximately one-third of all SBO cases nationwide. This enhances the cohort’s representativeness and improves the external validity and generalizability of our findings.

Several limitations should also be acknowledged. First, this was a pilot study with a relatively small sample size, which limits the statistical power to detect smaller differences between groups and increases the risk of type II error. The study did not capture exact emergency department waiting times or time to initiation of conservative management, which may introduce unmeasured variability between patients. Radiological predictors of small bowel obstruction severity were not systematically recorded or included in the analysis. Imaging interpretation was based on routine clinical assessment by experienced radiologists, but specific variables were not collected in a standardised manner. The decision to proceed to surgery was not fully protocolised and relied on clinician judgment, which may introduce inter-operator variability. Also, surgeon preference may have influenced the choice of operative approach and the decision to convert to laparotomy, potentially introducing performance-related variability. Although not statistically significant, there was an imbalance in the proportion of patients with a virgin abdomen between groups, with a higher number in the early administration group. This subgroup may differ in underlying etiology and clinical course compared to adhesive SBO, which could introduce confounding and influence outcomes. The number of prior SBO episodes was not systematically recorded, which limits more detailed analysis of recurrence patterns. The study focused primarily on early clinical outcomes, and longer-term outcomes, such as recurrent SBO, were not assessed. Age and symptom duration were dichotomised to improve model stability in a small cohort, although this may reduce statistical sensitivity.

## 5. Conclusions

This prospective randomised pilot study supports the safety and feasibility of structured conservative management for small bowel obstruction.

Furthermore, the timing of contrast media administration did not appear to adversely affect clinical outcomes. However, these findings should be interpreted with caution, as the study was not powered to detect modest but clinically meaningful differences between strategies. Larger, adequately powered multicentre studies are warranted to further define the optimal timing of CM administration. In addition, comparative studies between hyperosmolar and isotonic contrast agents are needed to clarify potential differences in therapeutic effect, safety profile, and overall clinical benefit in the management of small bowel obstruction.

## Figures and Tables

**Table 1:** Comparison of the study groups.

Patient Data	4 h Group *n* = 63	24 h Group *n* = 65	*p*-Value
Age, years (LQ; UQ)	68 (59; 79)	69 (59; 77)	0.836 ^e^
Sex:			0.356 ^f^
female	39 (61.9)	30 (46.2)	
male (%)	24 (38.1)	35 (53.8)	
Time from onset of symptoms, hours (LQ; UQ)	16 (3; 125)	18 (2; 120)	0.878 ^e^
WBC, 10^9^/L (LQ; UQ) ^a^	9.9 (2.2; 20.0)	8.6 (2.0; 17.5)	0.598 ^e^
CRP, mg/L (LQ; UQ) ^a^	5 (2.0; 15.0)	2 (2.0; 14.5)	0.782 ^e^
Previous history of SBO, *n* (%) ^b^	30 (47.6)	24 (35.9)	0.220 ^f^
Previously operated due to SBO, *n* (%)	13 (20.6)	8 (12.3)	0.203 ^f^
Previous number of operations (%)			0.200 ^f^
No operations	10 (15.9)	3 (4.6)	
1 operation	19 (30.2)	23 (35.4)	
2 operations	14 (22.2)	18 (27.7)	
≥3 operations	20 (31.7)	21 (32.3)	
Type of previous surgery, *n* (%)			0.703 ^f^
Open ^c^	43 (81.1)	46 (74.2)	
Laparoscopic	2 (3.8)	6 (9.7)	
Open + laparoscopic ^d^	8 (15.1)	10 (16.1)	
Median length of stay, days (LQ; UQ)	3 (2; 5)	3 (3; 5)	0.215 ^e^

LQ—lower quartile; UQ—upper quartile; SBO—small bowel obstruction; WBC—white blood cell count; CRP—C-reactive protein. ^a^ Blood tests taken at admission; ^b^ History of a previous SBO episode; ^c^ Laparotomy; open hernia repair; appendectomy, etc.; ^d^ History of both laparoscopic and open surgery; ^e^ Mann–Whitney test; ^f^ Chi-square test.

**Table 2:** Surgical details for operated patients.

Variable	4 h Group (*n* = 63)	24 h Group (*n* = 65)
Median number of radiographs during CM challenge	2	2
Patients undergoing surgery (%)	16 (25.4)	17 (26.2)
Viable bowel	8	8
Reversible changes	6	7
Bowel necrosis	2	2
Laparotomy	7	7
Laparoscopic surgery	6	6
Conversion to laparotomy	3	4

CM—contrast media.

**Table 3:** Univariable and multivariable logistic regression analysis predicting successful conservative treatment.

Variable	Univariable OR (95% CI)	Multivariable OR (95% CI)
CM administration at 24 h	1.04 (0.47; 2.30)	1.12 (0.49; 2.60)
History of previous SBO	2.9 (1.20; 7.16)	3.06 (1.23; 7.64)
Duration of symptoms < 24 h	1.86 (0.82; 4.22)	2.07 (0.87; 4.93)
Age > 75 years	1.37 (0.56; 3.40)	1.64 (0.63; 4.31)
Male sex	0.99 (0.44; 2.20)	0.96 (0.41; 2.22)

CM—contrast media; SBO—small bowel obstruction; OR—odds ratio; CI—confidence interval.

## Data Availability

All data generated or analyzed during this study are included in this article. Further enquiries can be directed to the corresponding author.

## Abbreviations

The following abbreviations are used in this manuscript:

SBO	small bowel obstruction
WSES	World Society of Emergency Surgery
CM	contrast media
WSCM	water-soluble contrast media
ASBO	adhesive small bowel obstruction
VA-SBO	virgin abdomen small bowel obstruction
CT	computed tomography
ICD	International Classification of Diseases
LQ	lower quartile
UQ	upper quartile
WBC	white blood cell count
CRP	C-reactive protein
eGFR	estimated glomerular filtration rate
